# Association of marital status with affective temperament, education, occupation, and bonding style

**DOI:** 10.3389/fpubh.2026.1785190

**Published:** 2026-06-04

**Authors:** Hirofumi Hirakawa, Takeshi Terao, Kentaro Kohno, Akari Sakai, Nobuko Kawano

**Affiliations:** 1 Department of Neuropsychiatry, Faculty of Medicine, Oita University, Yufu, Oita, Japan; 2 Oita Occupational Health Management Center, Nishinihon Occupational Health Center, Oita, Oita, Japan; 3 Department of Psychology, Faculty of Welfare and Health Science, Oita University, Oita, Oita, Japan

**Keywords:** affective temperament, education, occupation, bonding style, marital status

## Abstract

**Background:**

Marital status, such as marriage and divorce, is an important aspect of one's life and is associated with biopsychosocial and economic factors. The present study aimed to investigate the association of marital status with affective temperament, education, occupation, and bonding style.

**Methods:**

The dataset included 112 individuals consisting of 28 unmarried, 69 married, and 15 divorced individuals. First, demographic data, including age, gender, education, occupation, affective temperament, and bonding style, were compared among the three groups (unmarried, married, and divorced) using analysis of variance or χ^2^ test. Second, multinominal logistic regression analyses were performed.

**Results:**

The proportion of full-time employees was significantly lower than that of homemakers in married participants compared with divorced participants, whereas neither education, affective temperament, nor parental bonding was significantly associated with marital status.

**Conclusion:**

The present findings suggest that the proportion of full-time employees may be significantly lower than that of homemakers in married participants compared with divorced participants. Further prospective studies are needed to clarify causality and additional contributing factors.

## 1 Introduction

Marital status, such as marriage and divorce, is an important aspect in one's life that is associated with biopsychosocial and economic factors. Moreover, marital status depends on one's thoughts and values and, as such, has existential significance. Although several epidemiological studies have reported the same finding: marriage is good for health, and divorce threatens mental and physical wellbeing (1, 2), closer examination reveals more nuanced results (3). Indeed, turbulent marriages can breed depression and impair health, while marital closeness and happiness can also pose risks when couples share unhealthy behaviors (4). Separation and divorce may leave lasting scars on a small minority; however, most people recover their health and wellbeing quickly (5).

A recent review (6) on patients with cardiovascular disease showed that patients who are unmarried, including those who are divorced, separated, widowed, or never married, have an increased rate of adverse cardiovascular events when compared to their married

counterparts. Some studies suggest that marriage may have a more protective role for males than for females (6). Furthermore, dissatisfaction in a marriage and marriage quality have a significant impact on cardiovascular risk (6).

Although the study (7) is relatively old, it showed that both marriage protection effects (beneficial effects of marriage which reduce mortality and morbidity) and marriage selection effects (healthier individuals are more likely to get married and perhaps also more likely to stay married) contributed to the marital status for women who were not employed, whereas neither marriage protection effects nor marriage selection effects were observed for women who were employed full-time (7). Therefore, occupation status may be interacted with marital status.

In any case, how can marital status be determined? What factors influence decisions about marriage or divorce? This is an important issue of mental health. We hypothesized that individuals with certain affective temperament, specific parental bonding patterns in childhood, higher education in adolescence, and full-time job may have greater self-confidence and independence, potentially leading to remaining single (unmarried) or avoiding divorce due to greater resilience in overcoming marital hardships. The present study aimed to investigate this hypothesis.

## 2 Subjects and methods

### 2.1 Subjects

We used data from a previous study (8) on psychotherapy in apparently healthy participants. In the previous study, the inclusion criterion was individuals aged ≥ 20 years who provided written informed consent. Participants were recruited via electronic bulletin boards, physical bulletin boards, and flyers. Individuals with serious psychiatric disorders, as determined by the mini-international neuropsychiatric interview (M.I.N.I.), were excluded. Participants were recruited from Oct 1, 2016, and June 30, 2018. The Institutional Review Board of Oita University Faculty of Medicine approved the trial on Sep 14, 2016 (number B16-023). All participants provided written informed consent. In this opt-out study, we used the data from all 130 healthy participants in the dataset (8), which included 108 females and 22 males, with a mean age of 49.3 years (SD = 12.1). This opt-out study was approved by the ethics committee of the Oita University Faculty of Medicine on June 12, 2023 (approval number 2536).

### 2.2 Marital status

The dataset included the following categories (N: number of individuals): (1) unmarried (*N* = 35), (2) married (*N* = 74), (3) divorced (*N* = 15), (4) widowed (*N* = 3), (5) divorced and remarried (*N* = 2), and (6) no response (*N* = 1). We excluded categories 4 and 5 due to their small sample sizes, and category 6 because it did not fit the purpose of this study. Consequently, there were 124 participants.

### 2.3 Occupational status

Of the 124 participants, the distribution by working-time occupation category was as follows: (1) full-time employees (*N* = 67), (2) part-time employees (*N* = 17), (3) self-employed (*N* = 8), (4) homemakers (*N* = 28), and (5) university students (*N* = 4). We excluded categories 3 and 5 due to their small sample sizes. As a result, a total of 112 participants remained. The mean age was 49.6 (SD = 11.8) and the sex ratios (female:male) was 95: 17. These 112 participants were the primary analytic sample.

### 2.4 Affective temperament

Participants completed the Japanese version of the Temperament Evaluation of Memphis, Pisa, Paris, and the San Diego-auto questionnaire (TEMPS-A), a 110-item true–false questionnaire that assesses five temperament dimensions: depressive, cyclothymic, hyperthymic, irritable, and anxious (9). The TEMPS-A has been translated into Japanese, and the reliability and validity of the Japanese version have been established (10).

### 2.5 Education level

Of the 112 individuals, education levels were categorized as: (1) high school (*N* = 16), (2) college (*N* = 43), or (3) university or more (*N* = 53).

### 2.6 Bonding style

The dataset used for our previous study (8) contained data from the Parental Bonding Instrument (PBI), which is a retrospective self-assessment measure of parental rearing attitudes experienced in childhood (11). The PBI consists of two subscales of “care” and “overprotection.” A high score for “care” indicates more appropriate rearing (less rejection and indifference), whereas a high score for “overprotection” indicates inappropriate overprotection (less encouragement of self-reliance). The PBI consists of 25 items (12 items for care and 13 items for overprotection), and each item is assessed on a 4-point Likert scale. The PBI has been translated into Japanese, and the reliability and validity of the Japanese version have been established (12, 13).

### 2.7 Data analysis

First, our dataset contained missing values, which were retained as observed and were not replaced or imputed. Second, analysis of variance (ANOVA) or χ^2^ test was used to compare the demographic data (age, gender, education, occupation, affective temperament, and bonding style) across the three marital groups. Third, multinomial logistic regression analyses were conducted with marital category as the dependent variable and age, gender, education, occupation, affective temperament, bonding style as independent variables following the hypothesis. As a *post-hoc* comparison, our model used category 3 (divorced) as a reference group.

## 3 Results

The demographic data of primary analytic sample (*N* = 112) was shown in the Table 1. The number of missing data was shown in Tables 1, 2.

**Table 1:** Demographic data of the primary analytic sample (*N* =112).

Demographic variables	N	Min	Max	Mean	SD
Age	112	25	77	49.6	11.8
Gender	112	Female:Male = 95:17			
Depressive temperament	111 (missing 1)	0	16	8.7	3.5
Cyclothymic temperament	110 (missing 2)	0	18	5.0	4.3
Hyperthymic temperament	110 (missing 2)	0	18	4.8	3.4
Irritable temperament	110 (missing 2)	0	12	3.0	3.3
Anxious temperament	110 (missing 2)	0	24	6.9	5.5
Paternal care	107 (missing 5)	0	36	21.5	8.9
Paternal overprotection	107 (missing 5)	0	33	11.2	6.8
Maternal care	107 (missing 5)	0	36	24.5	7.8
Maternal overprotection	110 (missing 2)	0	36	11.4	7.4
Marital status	112	Unmarried:Married:Divorced = 28:69:15			
Educational status	112	High school:College:University or more = 16:43:53			
Occupational status	112	Full-time:Part-time:Homemaker = 67:17:28			

**Table 2:** Demographic data, including age, gender, education, occupation, affective temperament, and bonding style, across the three groups and results of ANOVA and χ^2^ test.

Demographic variables	Category^*^	*N*	Mean	SD	Min	Max	ANOVA or χ^2^ test
Age	1	28	42.2	13.2	25	77	F = 8.45 *p* < 0.0001
	2	69	51.9	10.6	31	77	
	3	15	53.1	8.6	40	72	
	Total	112	49.6	11.8	25	77	
Gender Female:Male	1	28	25:3				χ^2^= 0.74 *p* = 0.70
	2	69	57:12				
	3	15	13:2				
	Total	112	95:17				
Education High School:College:University or more	1	28	1:13:14				χ^2^= 8.85 *p* = 0.065
	2	69	11:22:36				
	3	15	4:8:3				
	Total	112	16:43:53				
Occupation Full-time:Part-time:Homemaker	1	28	22:2:4				χ^2^= 11.38 *p* = 0.023
	2	69	33:13:23				
	3	15	12:2:1				
	Total	112	67:17:28				
Depressive temperament	1	27 (missing 1)	8.9	3.1	3	15	F = 0.72 *p* = 0.49
	2	69	8.4	3.7	1	16	
	3	15	9.5	3.6	0	16	
	Total	111 (missing 1)	8.7	3.5	0	16	
Cyclothymic temperament	1	27 (missing 1)	7.0	5.6	0	18	F = 4.31 *p* = 0.016
	2	68 (missing 1)	4.5	3.8	0	16	
	3	15	3.7	2.9	0	10	
	Total	110 (missing 2)	5.0	4.3	0	18	
Hyperthymic temperament	1	27 (missing 1)	4.6	3.1	1	16	F = 0.098 *p* = 0.91
	2	68 (missing 1)	4.9	3.5	0	18	
	3	15	4.9	3.8	0	11	
	Total	110 (missing 2)	4.8	3.4	0	18	
Irritable temperament	1	27 (missing 1)	4.1	1.075	0	11	F = 1.98 *p* = 0.15
	2	68 (missing 1)	2.7	3.3	0	12	
	3	15	2.5	3.3	0	10	
	Total	110 (missing 2)	3.0	3.0	0	12	
Anxious temperament	1	26 (missing 2)	8.4	5.4	0	17	F = 1.16 *p* = 0.32
	2	69	6.5	5.5	0	24	
	3	15	6.3	5.4	0	15	
	Total	110 (missing 2)	6.9	5.5	0	24	
Paternal care	1	28	23.1	8.8	0	34	F = 0.84 *p* = 0.44
	2	65 (missing 4)	21.2	8.8	0	36	
	3	14 (missing 1)	19.5	10.1	3	35	
	Total	107 (missing 5)	21.5	8.9	0	36	
Paternal overprotection	1	28	9.5	4.5	1	17	F = 1.20 *p* = 0.31
	2	64 (missing 5)	11.8	6.9	0	32	
	3	15	12.0	9.3	0	33	
	Total	107 (missing 5)	11.2	6.8	0	33	
Maternal care	1	28	26.8	6.8	12	35	F = 3.71 *p* = 0.028
	2	65 (missing 4)	24.4	7.3	5	36	
	3	14 (missing 1)	20.0	10.2	6	36	
	Total	107 (missing 5)	24.5	7.8	5	36	
Maternal overprotection	1	28	11.5	6.3	1	28	F = 1.31 *p* = 0.28
	2	67 (missing 2)	10.8	7.3	0	27	
	3	15	14.2	9.4	3	36	
	Total	110 (missing 2)	11.4	7.4	0	36	

^*^Category: 1 (unmarried), 2 (married), 3 (divorced).

Table 2 shows the data of age, gender, education, occupation, affective temperament, and bonding style across the three groups along with the results of ANOVA or χ^2^ test.

Table 3 shows the results of multinominal logistic regression analysis for marital status as a dependent factor, with age, cyclothymic temperament, maternal care, gender (male, female), education (high school, college, and university or more), occupation (full-time, part-time, and homemaker) as independent factors, using category 3 (divorced) as a reference. The proportion of full-time employees was significantly lower than that of homemakers in category 2 (married) compared with category 3 (divorced). Neither education, cyclothymic temperament, nor maternal care was significantly associated with marital status.

**Table 3:** Multinominal logistic regression analysis for marital status with category 3 (divorced) as a reference.

Demographic variables	*P*	Exp(B)	Exp(B) 95% CI	
			Lower limit	Upper limit
1 (Unmarried)				
Constant	0.32			
Age	0.057	0.91	0.83	1.00
Cyclothymic temperament	0.34	1.11	0.90	1.39
Maternal care	0.15	1.08	0.98	1.19
Gender: male	0.51	2.40	0.18	32.44
Gender: female	Reference		
Education: high school	0.34	0.22	0.01	4.70
Education: college	0.49	0.49	0.06	3.74
Education: university or more	Reference		
Occupation: full-time	0.20	0.16	0.01	2.60
Occupation: part-time	0.27	0.17	0.007	3.90
Occupation: homemaker	Reference		
2 (Married)				
Constant	0.17			
Age	0.30	0.96	0.89	1.04
Cyclothymic temperament	0.88	1.02	0.83	1.25
Maternal care	0.22	1.06	0.97	1.15
Gender: male	0.90	1.17	0.12	11.63
Gender: female	Reference		
Education: high school	0.41	0.38	0.039	3.73
Education: college	0.11	0.22	0.034	1.41
Education: university or more	Reference		
Occupation: full-time	0.044	0.08	0.006	0.93
Occupation: part-time	0.33	0.26	0.018	3.74
Occupation: homemaker	Reference		

Reference: 3 (divorced).

## 4 Discussion

The findings of this study are that the proportion of full-time employees was significantly lower than that of homemakers in married participants compared with divorced participants, whereas neither education, affective temperament, nor parental bonding was significantly associated with marital status. Most of our hypothesis was not supported that individuals with certain affective temperament, specific parental bonding patterns in childhood, higher education in adolescence, and full-time job may have greater self-confidence and independence, potentially leading to remaining single (unmarried) or avoiding divorce due to greater resilience in overcoming marital hardships.

Unexpectedly, hyperthymic temperament was not associated with marital status. Although the reason is uncertain, hyperthymic temperament may be independent from any marital status. That is, hyperthymic temperament may have the ability to overcome any hardships during any marital status. Also, the other temperaments and parental bonding were not associated with marital status. These findings suggest that neither affective temperament nor parental bonding may not be associated with marital status. Although education was not associated with marital status, occupation was associated with marital status. Specifically, the proportion of full-time employees was significantly lower than that of homemakers among married participants compared to those who were divorced. Regrettably, it cannot support our hypothesis that individuals with full-time jobs possess greater self-confidence and independence, potentially helping them avoid divorce through greater resilience in overcoming marital hardships. Alternatively, this may be due to financial difficulties resulting from divorce or the necessity of homemakers in married households.

Interestingly, a recent study (14) showed that among the initially never married, those who became married had lower mortality, lower risks of cardiovascular diseases, greater psychological wellbeing and less psychological distress, whereas among the initially married, those who became divorced/separated had lower social integration, greater psychosocial distress, and possibly greater risks of mortality, cardiovascular diseases, and smoking. Further studies are warranted to investigate not only the predictors of marital status but also those of marital transitions.

This study had some limitations. First, this was a cross-sectional study, and causal relationships could not be determined. The observed associations are exploratory and cannot establish directionality or causal. Second, the sample size was relatively small and was heavily skewed toward middle-aged women, which precluded us from drawing any definitive conclusions. Finally, this study focused on only psychological and occupational aspects, and biological, social, economic, and cultural aspects should be considered sufficiently.

In conclusion, the present findings suggest that the proportion of full-time employees may be significantly lower than that of homemakers in married participants compared with divorced participants. Further prospective studies are needed to clarify causality and additional contributing factors.

## Data Availability

The data analyzed in this study is subject to the following licenses/restrictions: the datasets analyzed during the current study are available from the corresponding author on reasonable request. Requests to access these datasets should be directed to Takeshi Terao, terao@oita-u.ac.jp.
